# Personalized nursing interventions based on risk factors for post-traumatic stress disorder following intracerebral hemorrhage: an analysis of effectiveness

**DOI:** 10.3389/fpsyt.2025.1648588

**Published:** 2025-10-22

**Authors:** Chen Bu, Qinyun Zhang, Liang Xu

**Affiliations:** Department of Neurosurgery, Affiliated Hospital of Jiangnan University, Wuxi, China

**Keywords:** intracerebral hemorrhage, post-traumatic stress disorder, personalizednursing, risk factors, analysis of effectiveness

## Abstract

**Background:**

This study aimed to identify independent risk factors for post-traumatic stress disorder (PTSD) in intracerebral hemorrhage (ICH) patients and to assess the clinical effectiveness of personalized nursing interventions tailored to these risk factors.

**Methods:**

Ninety-one ICH patients with PTSD and 76 without PTSD admitted from January 2023 to January 2025 were included in the study. A retrospective analysis was performed to determine the factors associated with the development of PTSD in ICH patients and to develop targeted and individualized care strategies. Subsequently, a prospective cohort of 98 consecutive patients with ICH and PTSD were randomly assigned to either a targeted care group (n=49) or a usual care group (n=49). After 7 patients were lost to follow-up in the targeted care group, 42 patients in the observation group and 49 in the control group were included in the final analysis. Outcomes were measured using the Barthel Index (BI), Connor-Davidson Resilience Scale (CD-RISC), and SF-36 quality of life assessment.

**Results:**

Univariate analysis revealed a higher proportion of patients with hemorrhage involving the amygdala, hippocampus, or prefrontal cortex (P<0.05), as well as younger age, lower education, and poorer baseline CD-RISC and BI scores in the PTSD group. Multivariate regression analysis confirmed that brain region injury, low education level, low CD-RISC score, and low BI were independent risk factors for PTSD (P<0.05). The observation group (n=42) demonstrated superior outcomes in psychological resilience (CD-RISC), functional independence in daily living (BI), and quality of life (SF-36 physical function, social function, role emotional domains; all P<0.05). Patient satisfaction was significantly higher in the observation group (90.48%, P<0.05).

**Conclusion:**

PTSD following ICH is strongly associated with specific neuroanatomical damage, limited education, impaired psychological resilience, and functional disability. Our biopsychosocial model-based personalized nursing protocol effectively enhances psychological resilience, daily functioning, and overall quality of life in this patient population.

## Introduction

Intracerebral hemorrhage (ICH), a life-threatening subtype of acute cerebrovascular disease, carries substantial risks of disability and mortality, with survivors frequently suffering persistent neurological deficits including motor impairment and cognitive dysfunction (1). While advances in acute care have markedly improved survival rates in recent years (2), growing clinical attention has shifted to the underrecognized mental health consequences during rehabilitation, particularly post-traumatic stress disorder (PTSD). Current epidemiological data reveal that 23%-38% of ICH survivors develop PTSD within 3–6 months post-ictus, exhibiting characteristic symptom clusters of re-experiencing, hyperarousal, and emotional numbing that profoundly compromise functional recovery and quality of life (3). Despite these compelling findings, the predominant research remains disproportionately focused on acute management and physical rehabilitation, creating a critical gap in evidence-based strategies for psychological sequelae (particularly PTSD) mitigation (4, 5). This glaring disparity underscores the imperative for developing targeted PTSD interventions.

The existing risk factor literature presents notable limitations, with conventional analyses overemphasizing biomedical parameters (e.g., hematoma volume, lesion site) while neglecting psychosocial determinants such as individual psychological traits, social support systems, and illness perception (6, 7). Notably, clinical observations reveal significant heterogeneity among patient populations in stress response patterns, psychological resilience levels, and coping strategies, suggesting that a one-size-fits-all nursing model fails to meet individualized needs (8). To address these shortcomings, our investigation employs an integrative biopsychosocial approach to comprehensively elucidate the multidimensional predictors of PTSD pathogenesis in ICH survivors, thereby improving clinical understanding of PTSD progression and enabling more precise risk stratification.

Beyond theoretical contributions, this study pioneers a paradigm shift in neurorehabilitation by developing and validating a personalized care algorithm based on individualized risk profiling. Through a prospective cohort study, the clinical effectiveness of personalized care in reducing PTSD incidence, mitigating psychological stress responses, and enhancing rehabilitation adherence was evaluated, providing empirical evidence for establishing a comprehensive mental health management system for ICH patients.

The implications of these findings extend beyond addressing critical knowledge gaps in post-ICH psychological sequelae management. By operationalizing research insights into clinically actionable tools—including validated assessment instruments and customizable intervention modules—this work facilitates immediate implementation in real-world rehabilitation settings. Such translational outcomes promise to substantially enhance the quality and comprehensiveness of care for ICH survivors throughout their recovery trajectory.

## Materials and methods

### Study population

**Figure 1:**
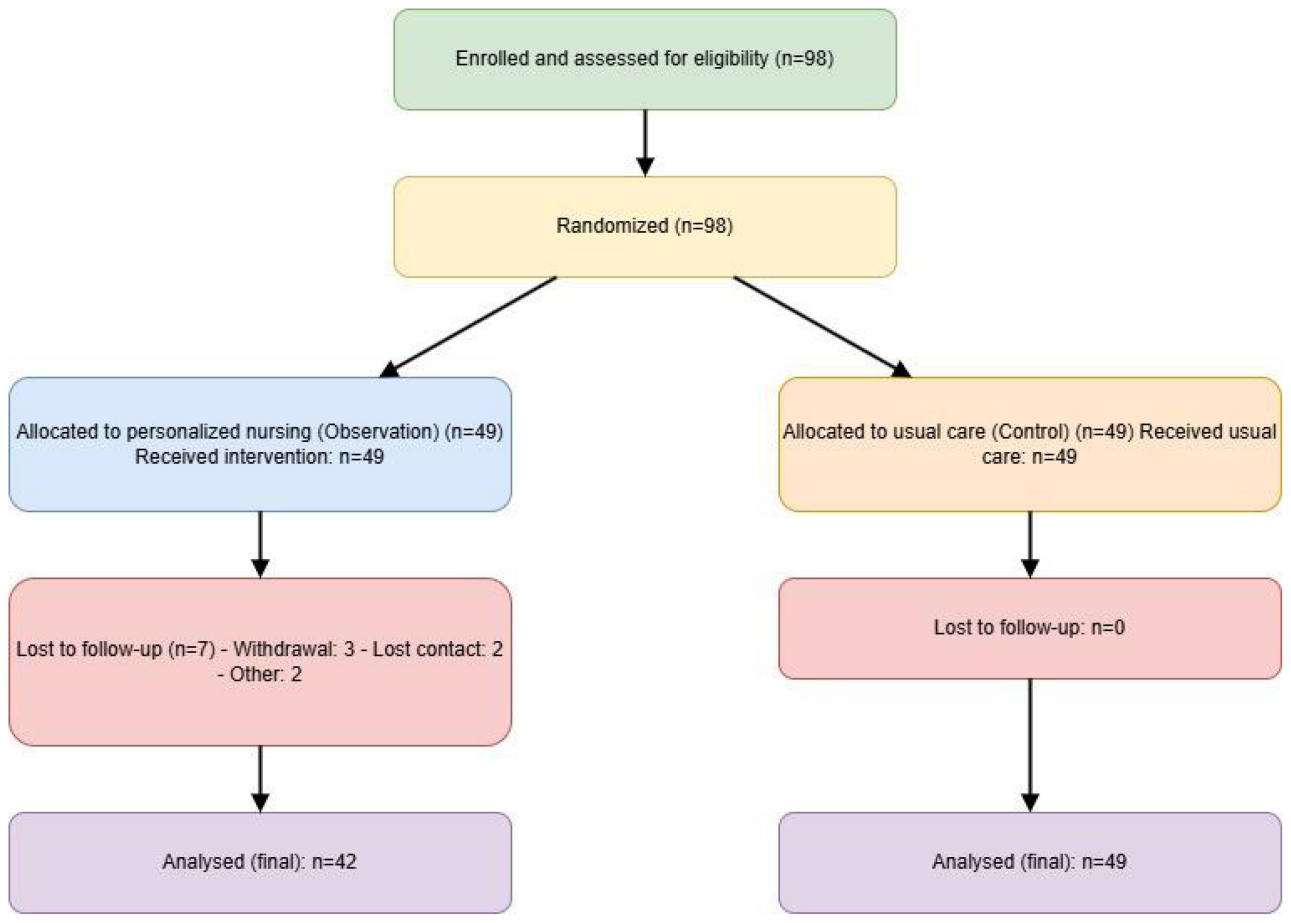
CONSORT flow diagram.

Firstly, a retrospective case-control analysis was conducted, including 91 ICH patients with PTSD and 76 without PTSD admitted between January 2023 and January 2025, to identify risk factors for PTSD development. Subsequently, a prospective interventional study was performed, in which a new cohort of 98 consecutive patients diagnosed with ICH and PTSD was enrolled (between January 2023 and December 2024) to evaluate the effectiveness of personalized nursing strategies. These participants were randomly assigned using a block randomization method (block size of 4) to either an observation group (personalized nursing interventions, n=49) or a control group (usual care, n=49). The randomization sequence was generated by an independent statistician using the sealed envelope method to ensure allocation concealment. During the 8-week intervention period, 7 patients in the observation group were lost to follow-up (due to withdrawal of consent [n=3], loss of contact [n=2], and other reasons [n=2]), while no losses occurred in the control group. Consequently, the final analysis included 42 patients in the observation group and 49 in the control group (total n=91). The participant flow is summarized in **Figure 1** (CONSORT diagram). Data analysis was performed based on the intention-to-treat (ITT) principle, with the last observation carried forward for the 7 patients lost to follow-up.

Due to the nature of the nursing interventions, complete blinding of participants and caregivers was not feasible. However, neither the outcome assessors nor the data analysts were privy to the grouping of patients throughout the study.

Sample size calculations were performed using G*Power software to ensure adequate statistical power. Written informed consent was obtained from all participants, and the study protocol was approved by the Institutional Review Board of Affiliated Hospital of Jiangnan University.

### Inclusion and exclusion criteria

Inclusion criteria: Absence of pre-existing cognitive impairment; Diagnosis of ICH confirmed by cranial CT imaging with hemorrhage volume <10 mL (9); Age ≥60 years with complete medical records; PTSD diagnosis meeting diagnostic criteria (10). Exclusion criteria: Comorbid severe organ dysfunction (cardiac, hepatic, or renal); Pre-existing psychiatric disorders; Exposure to additional traumatic stressors during the study period.

### Methods of nursing

Conventional Nursing: In accordance with the patient’s clinical condition, the bed head was elevated to an angle of 15°–30° to facilitate neck extension. In addition, the ward environment was maintained with adequate ventilation and lighting, while family visitation frequency was appropriately regulated. Medication was administered as prescribed, and a comfortable therapeutic setting was ensured throughout the treatment period. Furthermore, any clinical abnormalities were promptly reported to the attending physician for further evaluation and intervention.

Nursing strategies for PTSD risk factors: To address the risk factors of PTSD, we developed the following nursing strategies:

1. Exposure therapy and relaxation training in cognitive behavioral therapy (CBT) were used for 8 weeks. Exposure therapy: start with low-intensity stimulation (e.g., viewing neutral pictures), 2 times/week, 30 min/session, and gradually transition to high-intensity stimulation (e.g., trauma-related scene simulation). Relaxation training: deep breathing (5min) and progressive muscle relaxation (10min) 1 times/d in the morning and 1 times/d in the evening, guided by a nurse during hospitalization and tracked via an app after discharge.
2. Memory aid with structured schedule. A notebook was used to record the day’s schedule (10min) in the morning of each day, and patients were reminded to check the completion of tasks every 2h. The patient’s schedule was developed by the rehabilitation therapist to include fixed time points (e.g., 9:00 for medication, 14:00 for rehabilitation training) and adjusted once a week according to the patient’s adaptation.
3. Task planning and decision-making training. Choose 1 complex task (e.g., preparing breakfast) every day and complete it in 3 steps (planning→execution→review), 20 min each time, 5 times/week. Training through scenario cards (e.g., “how to deal with sudden power outage”), 2 times/week, 30min/time.
4. For patients with a low level of education, a copy of the booklet (containing illustrations of medication and cartoons of rehabilitation steps) is distributed after each medical consultation, and key points are marked with a red pen. A short 10-min video (with dialect dubbing) covering the effects of medication and rehabilitation contraindications was shown once a week. Family members were asked to repeat key information (e.g., “medication should be taken after meals”) on a daily basis, and nurses randomly checked the information 2 times/week. Nursing training was provided to the patient’s family, with 3 sessions (1h each) covering first aid skills and emotional calming techniques during the first week of admission. 2 practice drills (e.g., simulating the patient’s emotional breakdown scenario) were conducted 1 week before discharge, each 45 min.
5. Staged exercise rehabilitation for patients with low CD-RISC scores and BI. It includes passive training (bed joint mobilization, 2 times/d, 20min/time, assisted by a rehabilitator) and active training (seated balance, 1 time/d (10min/time) during the first week, and then increased to 2 times/d, 15min/time from the second week. Walking training, using a walker, 3 times/d, gradually extended from 5min/trip to 20min/trip). Another was given to design cognitive training games, including memory card game (1 times/d, 15min/repetition, with the goal of correctly matching 10 sets of cards for 3 consecutive days), number sequencing task (3 times/week, 10min/repetition, patients were asked to sequentially arrange the numbers from 1-20), and simulation of daily tasks (e.g., simulation of shopping in supermarkets, 2 times/week, 30min/repetition, and 5min replay after completion).

### Questionnaire survey

Patient assessments were conducted using the Barthel Index (BI) (11) and the Connor-Davidson Resilience Scale (CD-RISC) (12), with higher scores indicating better functional independence in daily living and psychological resilience, respectively. Following the implementation of PTSD-specific nursing interventions, patient recovery was evaluated using the Short Form-36-item Health Survey (SF-36) (13). Additionally, upon discharge, nursing satisfaction was assessed via a structured questionnaire with a maximum score of 75. Satisfaction levels were categorized as follows: <60: unsatisfied; 60–70: moderately satisfied; >70: satisfied. The overall satisfaction rate was calculated as: (satisfied + moderately satisfied)/total number of patients ×100%.

### Statistical analysis

Data were analyzed using SPSS 25.0. Categorical variables were expressed as frequencies (%) and compared using the chi-square test. Continuous variables were presented as mean ± standard deviation (`χ ± s) and analyzed via independent samples t-tests. Logistic regression analysis was employed to identify potential influencing factors. A P-value < 0.05 was considered statistically significant.

## Results

### Univariate analysis of factors influencing PTSD after ICH

Univariate analysis of factors influencing PTSD after ICH showed that demographic characteristics including sex and residential area did not differ significantly between PTSD and non-PTSD groups (P > 0.05). Notably, the PTSD group demonstrated a significantly higher prevalence of hemorrhagic involvement in the amygdala, hippocampus, and prefrontal cortex compared to non-PTSD controls. Furthermore, patients with PTSD were younger, had lower educational levels, and scored significantly lower on both the CD-RISC and BI assessments (P < 0.05). Importantly, the PTSD group exhibited a significantly larger ICH volume compared to the non-PTSD group (P < 0.001; **Table 1**).

**Table 1:** Univariate analysis of factors influencing ptsd after ICH.

Projects	Non-PTSD patients (n=76)	PTSD patients (n=91)	t (χ^2^)	P
Age	67.29 ± 4.51	66.01 ± 3.16	**2.147**	**0.033**
Sex			0.329	0.566
male	46 (60.53)	59 (64.84)		
female	30 (39.47)	32 (35.16)		
Educational levels			**5.976**	**0.015**
junior high school and below	29 (38.16)	52 (57.14)		
high school and above	47 (61.84)	39 (42.86)		
Residence			2.204	0.138
live alone	6 (7.89)	14 (15.38)		
living with family	70 (92.11)	77 (84.62)		
Place of residence			1.063	0.303
urban	34 (44.74)	48 (52.75)		
rural	42 (55.26)	43 (47.26)		
Family history of disease			1.375	0.241
yes	19 (25.00)	16 (17.58)		
no	57 (75.00)	75 (82.42)		
Comorbidities				
diabetes	34 (44.74)	42 (46.15)	0.034	0.855
hypertension	56 (73.68)	64 (70.33)	0.230	0.631
hyperlipidemia	17 (22.37)	24 (26.37)	0.359	0.549
Smoking			0.561	0.454
yes	34 (44.74)	46 (50.55)		
no	42 (55.26)	45 (49.45)		
Alcohol drinking			2.684	0.101
yes	24 (31.58)	40 (43.96)		
no	52 (68.42)	51 (56.04)		
ICH Volume (mL)	7.99 ± 0.62	8.38 ± 0.62	-4.049	**<0.001**
Bleeding involving amygdala/hippocampus/prefrontal cortex			**12.553**	**<0.001**
yes	31 (40.79)	62 (68.13)		
no	45 (59.21)	29 (31.87)		
CD-RISC score	77.66 ± 7.72	69.97 ± 8.61	**6.021**	**<0.001**
BI	75.45 ± 8.01	67.43 ± 10.29	**5.353**	**<0.001**

Bold values indicate that the difference is statistically significant at p < 0.05.

### Multivariate analysis of factors influencing post-ICH PTSD

A multivariate logistic regression analysis was performed with PTSD occurrence as the dependent variable, incorporating the statistically significant indicators from the univariate analysis as covariates (Non-PTSD=1, PTSD = 2; high school and above=1, junior high school and below=2; non-bleeding involving amygdala/hippocampus/prefrontal cortex=1, bleeding=2). The results demonstrated that age (OR = 0.901, 95% CI = 0.818–0.993, P = 0.035), educational level (OR = 2.139, 95% CI = 1.025–4.461, P = 0.043), bleeding involving amygdala/hippocampus/prefrontal cortex (OR = 0.360, 95% CI = 0.171–0.758, P = 0.007), CD-RISC score (OR = 0.898, 95% CI = 0.855–0.943, P<0.001), and BI score (OR = 1.059, 95% CI = 1.011–1.110, P = 0.016) were independent factors influencing PTSD after ICH. ICH volume was not significantly associated with PTSD in the multivariate model (OR = 1.107, 95% CI = 0.612–2.002, P = 0.736) (**Table 2**).

**Table 2:** Multivariate analysis of factors influencing post-ICH PTSD.

Projects	β	S.E.	Wals	P	OR	95%CI
Age	-0.104	0.049	4.455	0.035	0.901	0.818-0.993
Educational levels	0.76	0.375	4.106	0.043	2.139	1.025-4.461
Bleeding involving amygdala/hippocampus/prefrontal cortex	-1.021	0.38	7.231	0.007	0.36	0.171-0.758
CD-RISC score	-0.108	0.025	18.673	0	0.898	0.855-0.943
BI	0.057	0.024	5.808	0.016	1.059	1.011-1.110
ICH Volume	0.102	0.302	0.114	0.736	1.107	0.612-2.002

β, regression coefficient; S.E., standard error; OR, odds ratio; 95%CI, 95% confidence interval.

### Clinical outcomes of tailored nursing intervention

**Figure 2:**
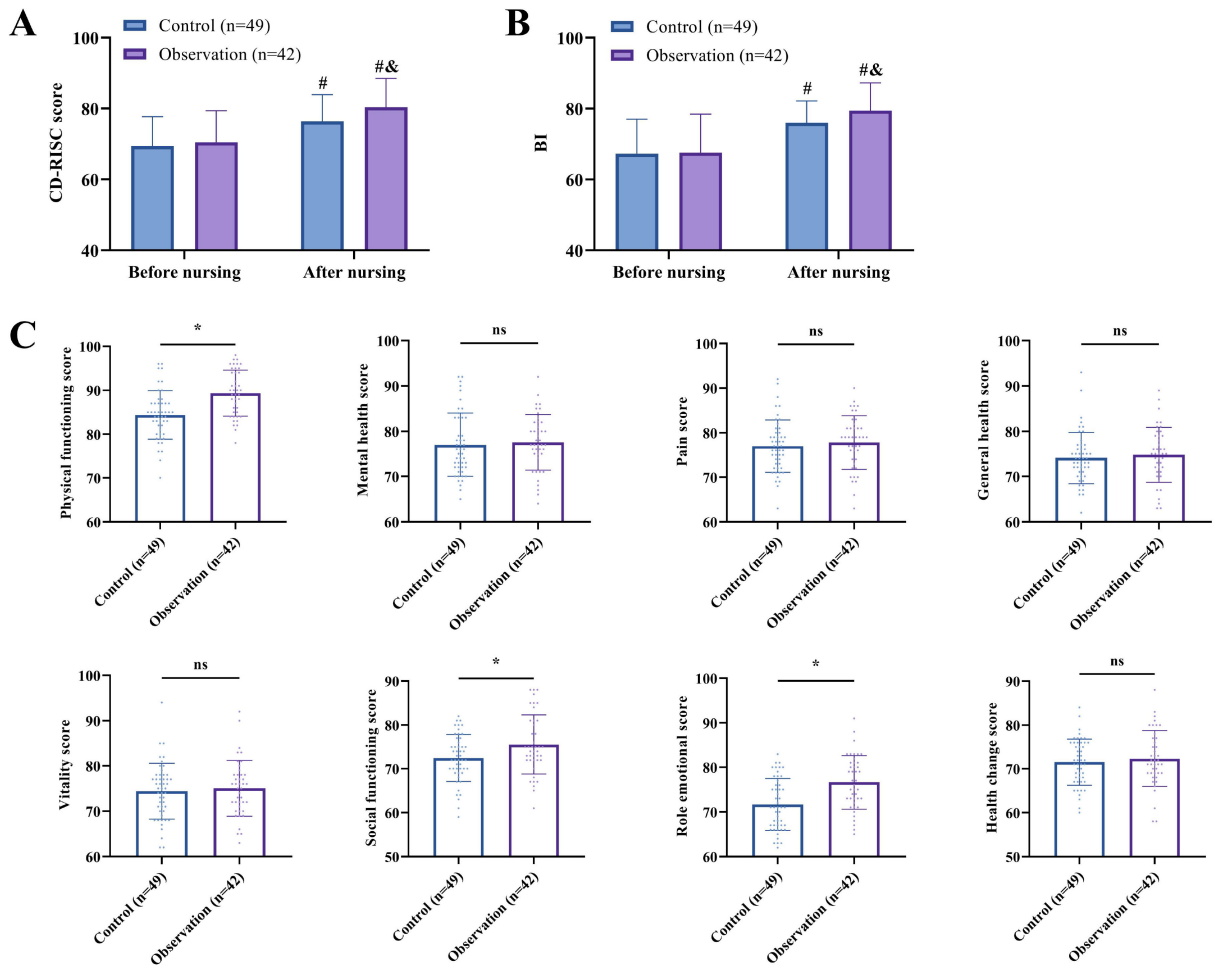
Effectiveness of the application of personalized care for PTSD. **(A)** Comparison of changes in CD-RISC score before and after targeted care. **(B)** Comparison of changes in BI before and after targeted care. **(C)** Comparison of SF-36 scores after targeted care. ^#^indicates P<0.05 compared to pre-care, & indicates P<0.05 compared to the control group, *indicates that the difference between the two groups is statistically significant (P<0.05), and ns indicates that the difference between the two groups is not statistically significant (P>0.05).

Prior to the intervention, baseline assessments revealed no statistically significant differences in CD-RISC score or BI between the two groups (P>0.05). Following the nursing intervention, both groups demonstrated significant improvements in psychological resilience (CD-RISC) and activities of daily living (BI), with the observation group exhibiting markedly superior outcomes compared to the control group (P<0.05). Furthermore, post-intervention SF-36 quality-of-life assessments indicated significantly higher scores in physical functioning, social functioning, and role-emotional domains among patients receiving tailored nursing care (P<0.05) (**Figure 2**).

### Enhanced patient satisfaction with tailored nursing approach

Patient satisfaction evaluations conducted post-intervention demonstrated a significantly higher overall satisfaction rate in the observation group (90.48%) compared to conventional care recipients (73.47%; P = 0.038) (**Table 3**).

**Table 3:** Comparison of satisfaction survey results.

Groups	Satisfied	Moderately satisfied	Unsatisfied	Overall satisfaction rate
Control group (n=49)	13 (26.53)	23 (46.94)	13 (26.53)	73.47%
Observation group (n=42)	18 (42.86)	20 (47.62)	4 (9.52)	90.48%
χ^2^				4.306
P				0.038

## Discussion

The main findings of this study are that hemorrhagic involvement of the amygdala, hippocampus, or prefrontal cortex, lower educational level, reduced psychological resilience (CD-RISC score), and impaired functional independence in daily living (BI score) are independent risk factors for the development of PTSD after ICH. Notably, although univariate analysis showed a larger ICH volume in the PTSD group, multivariate analysis indicated that ICH volume was not an independent predictor of PTSD. Furthermore, tailored nursing interventions based on these risk factors significantly improved psychological resilience, functional independence, quality of life, and patient satisfaction.

These findings are consistent with and extend previous literature. Neurobiological evidence supports the importance of lesion location: the amygdala plays a pivotal role in fear conditioning and emotional memory processing, the hippocampus participates in the encoding and retrieval of traumatic events, and the prefrontal cortex regulates emotion and cognitive control (14). Disruption of this neural circuitry through hemorrhagic injury may hinder traumatic memory extinction, thereby perpetuating PTSD symptoms (15), which aligns with our results. From a psychosocial perspective, lower education levels may heighten uncertainty regarding the condition due to limited health literacy and information-processing ability, thereby exacerbating anxiety and avoidance behaviors (16). Psychological resilience, as a core capacity for coping with stress, may diminish an individual’s ability to adapt to sudden traumatic events when impaired, which our results also corroborate. Additionally, impaired activities of daily living (BI scores) can worsen psychological distress by limiting social participation and functional recovery (17). These findings underscore the multifactorial etiology of post-ICH PTSD, highlighting the complex interplay between neuroanatomical damage, psychological vulnerability, and social determinants of health, which aligns with the biopsychosocial model of disease.

Importantly, univariate analysis indicated that patients with PTSD had significantly larger ICH volumes compared to those without PTSD. This observation is in line with prior evidence suggesting that greater hemorrhage burden may aggravate disruption of neural networks involved in emotion regulation and memory processing, and may also contribute to functional disability and dependence in daily living (18). However, in our multivariate analysis, ICH volume was not retained as an independent predictor, suggesting that while larger hematoma size may exert indirect effects through neurological and functional impairment, lesion location and psychosocial factors play a more decisive role in the development of post-ICH PTSD.

Our study further demonstrated the effectiveness of a tailored nursing intervention model that integrates neuropsychological principles and evidence-based strategies. For patients with structural or functional impairments in key emotion-regulating brain regions, we adopted a combined approach of CBT and compensatory memory strategies, supported by prospective clinical trials (19). Graded exposure therapy facilitates fear extinction through systematic desensitization to trauma-related cues, and structured activity scheduling mitigates executive dysfunction and anxiety secondary to memory impairment (20) (19). For patients with lower education levels, health education protocols adapted through simplified information delivery were employed to optimize disease self-management (21). The rehabilitation framework included progressive cognitive training to leverage neuroplastic mechanisms and foster psychological resilience via achievable task completion (22). Family empowerment was a key feature of the intervention, consistent with evidence that robust social support improves treatment adherence (23).

The observed superiority of the tailored intervention group in psychological resilience (CD-RISC), functional independence (BI), and quality-of-life measures (SF-36) supports the therapeutic value of this approach, consistent with previous research demonstrating the benefits of risk-stratified personalized care for PTSD (24). The significant improvement in patient satisfaction (90.48% vs. 73.47%) further confirms the feasibility and acceptability of this model in clinical practice. Mechanistically, these improvements likely stem from enhanced coping strategies that reduce PTSD symptom severity, restoration of functional capacity to disrupt the cycle of impairment and distress, and improved psychosocial functioning through patient-centered care (25).

### Limitations

This study has several limitations. First, its retrospective design may introduce selection bias, limiting the generalizability of the findings. Second, the modest sample size could reduce statistical power and limit the detection of smaller effect sizes. Third, the absence of long-term follow-up precludes conclusions regarding the sustained efficacy of the tailored intervention. Lastly, while we focused on clinical and neuropsychological predictors, additional biological markers such as gene expression profiles and inflammatory cytokines were not assessed, which could further refine risk stratification and intervention targeting. Future multicenter prospective studies with larger sample sizes and extended follow-up are warranted to validate these findings and optimize intervention strategies.

## Conclusion

Hemorrhage involving the amygdala, hippocampus, or prefrontal cortex, lower education level, reduced psychological resilience, and impaired functional independence are independent predictors of PTSD following ICH. Tailored nursing interventions addressing these risk factors significantly improve psychological resilience, functional independence, quality of life, and patient satisfaction. These findings provide evidence for the development of targeted, multidisciplinary strategies to prevent and manage PTSD in ICH patients, supporting their integration into clinical practice.

## Data Availability

The original contributions presented in the study are included in the article/supplementary material. further inquiries can be directed to the corresponding author.
